# ENHANCE—(Electronic Hydroxyurea Adherence): A Protocol to Increase Hydroxyurea Adherence in Patients with Sickle Cell Disease

**DOI:** 10.2196/resprot.6403

**Published:** 2016-10-03

**Authors:** Susan Creary, Deena J Chisolm, Sarah H O’Brien

**Affiliations:** ^1^ Nationwide Children's Hospital The Ohio State University School of Medicine Columbus, OH United States; ^2^ Nationwide Children's Hospital The Ohio State University Columbus, OH United States

**Keywords:** hydroxyurea, children, sickle cell disease, adherence intervention

## Abstract

**Background:**

Hydroxyurea (HU) is the only disease-modifying medication for patients with sickle cell disease (SCD). HU can reduce SCD-related complications but only 35% to 50% of pediatric patients adhere to HU at the rates achieved in clinical trials and this limits its clinical effectiveness. Mobile Directly Observed Therapy (Mobile DOT) is a pilot-tested, electronic, multidimensional, HU adherence intervention that targets many components of the Health Behavior Model.

**Objective:**

The aim of this study is to evaluate the impact of Mobile DOT on HU adherence in children with SCD. The objective of our study is to inform the development of future adherence interventions and pediatric SCD protocols.

**Methods:**

This is a single-arm crossover study of pediatric patients with SCD. Participants self-record videos of their daily HU administrations and receive text message alerts to take HU, feedback on their HU adherence, and incentives when they achieve adherence goals during the 6-month Mobile DOT phase. Participants’ HU adherence during the Mobile DOT phase is compared with their baseline HU adherence (6 months prior to study entry) and to their HU adherence 6 months after completing the Mobile DOT phase. The primary outcome of this study is HU adherence measured by medication possession ratio.

**Results:**

The trial is ongoing. Preliminary review of participant satisfaction results suggest that most participants can complete Mobile DOT in less than 5 minutes per day and are satisfied with the intervention.

**Conclusions:**

If effective, the Mobile DOT strategy will increase HU adherence and this could improve patients’ clinical outcomes and reduce costs of care.

## Introduction

Sickle cell disease (SCD) is a chronic, inherited, red blood cell disorder that affects approximately 28,000 children in the United States and leads to substantial morbidity, premature mortality, and annual health care costs of approximately $335 million [1-4]. Vaso-occlusive pain and acute chest syndrome episodes are the 2 most common SCD-related complications [2]. Hydroxyurea (HU) is the only disease-modifying medication for patients with SCD. HU is a once-daily medication taken by mouth that comes in a capsule or liquid formulation and clinical trials indicate that HU reduces the frequency of vaso-occlusive pain and acute chest syndrome episodes, mortality, and health care costs for pediatric patients [5-8]. In a randomized, controlled study of HU for pediatric patients with SCD, 90% of patients achieved ≥80% HU adherence [9]; however, studies that measure HU adherence in clinical practice suggest that only 35% to 50% of pediatric patients achieve this high adherence rate [10,11]. Children who have poor HU adherence also have worse health outcomes and increased health care costs compared with those who have high adherence [11], but targeted adherence interventions to increase HU adherence remain untested.

HU adherence in pediatric patients with SCD is challenging for multiple reasons. First, children with SCD in the United States are primarily African American. African American children are more likely to live in poverty than non-Hispanic White [12], and impoverished patients are more likely to face systematic medication adherence barriers, such as poor access to pharmacies and higher medication copays than those of higher economic status [13]. Second, HU is a chronic medication, not curative, and the clinical benefits may take months to manifest [14]. Third, children and adolescents with SCD are also a diverse patient population with age-specific adherence barriers. Young children rely on caregivers to administer their medications and may be uncooperative with their medication administration. Adolescents have developmental and psychosocial factors, such as failing to accept that they have a chronic disease that can reduce their adherence [15]. Parents of children with SCD report that dealing with competing responsibilities are a barrier to HU adherence and that receiving support and the positive impact of HU on their children’s health facilitate adherence [10]. Finally, it is difficult to determine which patients have poor HU adherence or if interventions are successful at increasing adherence because validated HU adherence measures (eg, self-reported adherence, biomarkers, and refill rates) do not exist for this population of patients or for this medication.

**Figure 1:**
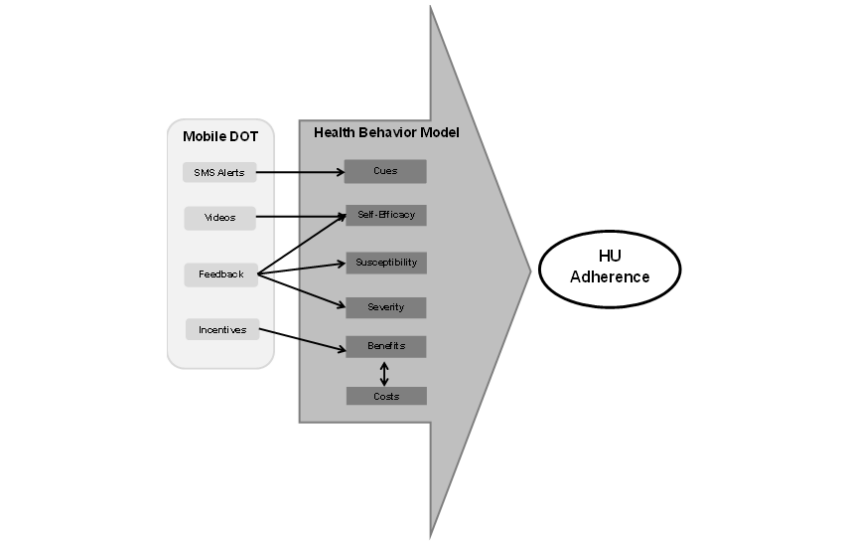
Mobile Directly Observed Therapy (Mobile DOT) and the health behavior model. HU: hydroxyurea; SMS: short message service.

The Health Behavior Model is a theoretical model commonly used to explain patients’ medication adherence behavior. The Health Behavior Model suggests that the variables that predict medication adherence behavior are patients’ perceived susceptibility, perceived disease severity, perceived benefits, perceived costs, cues to action, and self-efficacy (Figure 1) [16]. Perceived susceptibility is the level to which patients accept that they have a disease, and perceived disease severity is their valuation of whether that disease should receive treatment. Patients balance their perceived benefits of medication (eg, feeling better) with their perceived costs of a medication (eg, side effects). Cues remind patients to take medications and can be internal, such as experiencing a symptom that reminds them to take medication to alleviate that symptom, or external, such as receiving a prompt from another person to take medication. Finally, self-efficacy is patients’ confidence in their ability to adhere to a medication [17] and it can be influenced by patients’ prior experiences, their observations of others, and by the external input or support that they receive from others.

Many adherence interventions that target individual Health Behavior Model variables exist, but each has its limitations for use in children with SCD. For example, electronic alerts can provide cues to patients to prevent forgetting, but these devices can be expensive and they do not have the ability to remind, monitor, and encourage patients in a single application [18]. Electronic monitoring and providing feedback to patients is a feasible intervention that has been used in high risk, minority children with asthma, and can be an effective strategy because it serves to promote self-efficacy and also educate patients and parents about their disease severity and susceptibility [19-20], but it can be time consuming. Contingency management, or providing monetary incentives to patients for completing a healthy behavior [21-23], increases the benefit of being adherent to patients but can also be expensive and may not sustain adherence long-term [19]. Finally, directly observed therapy (DOT) involves health care workers traveling to observe patients ingest medications. It supports and encourages patients to administer their medications and it is the most successful medication adherence intervention for communicable diseases, including tuberculosis and human immunodeficiency virus [24,25]. However, it is unclear if DOT is effective for a noncommunicable disease or for chronic medications or if it would be cost-effective for these conditions or treatments long-term.

To overcome the multiple adherence barriers that exist for HU and children and adolescents with SCD, we created Mobile Directly Observed Therapy (Mobile DOT), an innovative, multidimensional adherence strategy. We pilot-tested Mobile DOT and found that a small population of children and adolescents with SCD could achieve ≥90% HU adherence using this strategy [26]. Adherence experts suggest that smartphone technology has the potential to improve medication adherence in children with chronic diseases because it takes advantage of a medium that youths already frequently use [27,28]. We designed Mobile DOT to target multiple Health Behavior Model variables using this widely available technology to deliver this multifaceted adherence approach without requiring additional software. Mobile DOT sends text message reminder alerts to cue patients to take HU. Patients record videos of their daily HU administrations to promote self-efficacy through experiential learning. Patients receive feedback from the research staff to increase self-efficacy and impact their perceived susceptibility to SCD-related complications and their perceived SCD severity. Finally, patients receive monetary incentives when they achieve adherence goals to increase the perceived benefits of taking HU (Figure 1).

The current study aims to determine if Mobile DOT increases HU adherence in children with SCD. This paper describes the study protocol in detail, the different components of the Mobile DOT intervention, and the measures that will be obtained. In addition, we will also determine if other measures (biomarkers, self-reported adherence, and refill adherence) are valid HU adherence measures. Finally, since adolescents with SCD are at particularly high risk of complications and death due to poor self-management skills at the time of transition to adult care [4,29], we will explore the impact of Mobile DOT on adolescents’ self-management skills. If effective, Mobile DOT has the potential to improve these patients’ health outcomes and significantly reduce their costs of care.

## Methods

### Study Design, Study Visits, and Data Collection

This is an 18-month, single-arm, crossover study that includes both young children and adolescents with SCD (Table 1). Participants’ receive 6 months of the Mobile DOT intervention. Their HU adherence during their Mobile DOT phase is compared with their HU adherence during the 6 months prior to enrollment (baseline), and their HU adherence during the 6 months after they receive Mobile DOT (observation). Study visits occur during standard of care monitoring visits and the visit windows are wide to increase this study’s feasibility and appeal and to prevent influencing patients’ adherence behavior with more frequent visits. Participants receive a US $25 gift card after they complete each study visit for the time they spent completing study surveys.

### Setting

Nationwide Children's Hospital (NCH) is a comprehensive, pediatric, tertiary care center. NCH provides care for approximately 380 patients, ages 0-21, with SCD. The principal investigator is a hematologist who provides clinical care for patients with SCD at NCH.

### Participants

All participants must provide informed consent to participate. Participants who are <18 years at enrollment are required to have their consenting caregiver provide consent, and participants 9-17 at study entry are required to provide assent. Eligibility criteria include the following: (1) age ≤19 years, (2) diagnosis of SCD (any genotype), (3) prescribed HU for at least the previous 6 months to allow time for patients to have achieved a stable HU dose, (4) planning to receive SCD-related care at NCH for the study duration, (5) participants ≥18 years must have personal daily access to a smartphone capable of recording and submitting videos to Mobile DOT, (6) consenting caregivers of participants <18 years must have daily access to a smartphone capable of recording and submitting videos to Mobile DOT and agree to participate in the participant’s HU administration routine, and (7) participants and/or the consenting caregivers must speak English. Patients who receive concurrent, chronic, red cell transfusion therapy (simple or exchange transfusion) are excluded because red cell transfusions affect HU biomarkers. Participants 16- to 19-years old at enrollment are considered adolescents. Participants are recruited using recruitment letters and approached when they present for care. If a prospective participant does not enroll on the study, the reason for why he or she did not enroll is recorded. The enrollment and withdrawal data are reviewed at least monthly during the study to ensure that recruitment and attrition goals are achieved and modification to these procedures are not required to achieve the aims of the study.

### Mobile DOT

Mobile DOT includes four aspects: reminder text message alerts, participant videos, feedback on adherence, and monetary incentives. Participants receive a Mobile DOT tutorial after enrollment to confirm that they receive the text message alerts, are able to send acceptable and viewable videos as an email attachment, and understand what is required to receive adherence incentives. Participants also receive a study sheet with information on what to do if they temporarily lose their smartphone and how to contact the research team if they have technical problems. Participants’ telephone numbers are confirmed at each study visit, and participants are responsible for notifying the research team if they change their telephone number or lose or break their smartphone. During the first 2 weeks of the study, the research team follows closely with participants (up to daily) by telephone, text message, or email, if necessary, to resolve any technical issues. The research staff also communicates with participants throughout the study to resolve technical issues by telephone, text message, email, or during appointments at NCH if participants notify the research team that they are having issues.

### Secure Mobile DOT Website

A secure Mobile DOT website was built by the research technology team at NCH to send the text message alerts and to receive, store, and view participant videos. The Web application runs under an Apache Web server (version 2.2.15; Apache Software Foundation), was written in the Perl and Javascript languages, and runs on a 64-bit CentOS 6.5 Linux operating system. A secure Microsoft Exchange mail server sends the automated text message alerts to the participants and the background scripts retrieve the emails with the video attachments. All participant videos are stored locally on the operating system in a directory with restricted access.  All information about the participants, the emails, and videos received from the participants are stored in a local SQLite (SQLite Consortium) database. 

### Text Message Reminder Alerts

Participants select their preferred HU medication administration time and create their own personalized text message reminder alerts at enrollment. For example, 2 participants selected, “It’s Go Time!” and “Reminder! It’s Meds Time” to be sent to remind them to take HU. Up to 4 of these alerts and one confirmation message (if a video is received) are sent to participants and/or the consenting caregivers each day. The alerts are discontinued for that day, once a video is received.

### Videos

Participants record their daily HU administration with their smartphone and deliver these videos to the secure website by attaching the video to an email, which they send to the secure server. Participants are informed during the consent process that there is a risk that these videos could potentially be intercepted during the delivery process but that they are secure once they are received by the website. This information is also included specifically in their informed consent document. Participants are instructed to take the HU dose prescribed by their provider, have their labeled HU medication bottle and dose ready when they begin recording their video, and that each video be brief, unique, and continuously recorded in high enough definition to allow for easy recognition of the participant and HU in its capsule or liquid formulation. They are trained so that the videos include the participant ingesting HU and the participant opening their mouth after they ingest HU. Participants are told to submit each video on the day that it was recorded, but videos that are received after that date are still considered valid if they are received within 7 days or if technical issue occurred.

Consenting caregivers are responsible for informing other supervising adults how to submit the videos if they are not available. In the unusual event that participants are instructed to temporarily discontinue HU by their SCD providers, participants are instructed to continue to submit daily videos stating this. Participants are not required to submit videos if they are seen in the emergency department. If they are hospitalized, their electronic medical administration records are used to confirm HU adherence. Temporary lapses (up to 5 days) in smartphone access are allowed, but participants must email, text message, or leave a voicemail for the research team each of these days to confirm that HU was taken.

### Adherence Feedback

The research staff observes all submitted videos within 72 hours of receipt and provides feedback to study participants to encourage HU adherence. This communication occurs after each missed video and after adherence goals are achieved (≥90% HU video adherence for 30 days). The research staff determines whether this feedback should occur via text message, email, or telephone call, so that it does not become intrusive and the type of communication that is used is documented.

### Incentives

Participants receive a US $30 gift card within 2 weeks if they achieve ≥90% video HU adherence for each 30-day period during the Mobile DOT phase of the study. Partial compensation is not provided. The 30-day study periods during the Mobile DOT phase are sequential, but participants who miss more than 3 videos early in a period can begin the next 30-day period early, if they submit at least 5 consecutive videos.

### Measures

#### Medication Possession Ratio

Participants sign a pharmacy release form at enrollment and the list of pharmacies that they use is reviewed with the research staff at each study visit. Participants’ HU refill records and hospitalization medication administration records are used to calculate their medication possession ratio (MPR) for each study phase using the following formula: MPR is the total number of days the participant had access to HU during the study phase divided by the total number of days that the participant was prescribed HU during the study phase.

#### Laboratory Data

Participants’ laboratory studies are obtained per NCH standard of care for patients prescribed HU. This monitoring includes measuring mean corpuscular volume (MCV) and fetal hemoglobin (HbF) at each monitoring visit. Prior studies show that HU induces HbF production and increases MCV [30,31], but it is unknown if these routinely obtained biomarkers are valid HU adherence measures. HbF at NCH is measured using Sebia Zone Electrophoresis and MCV is measured using a standard coulter counter.

#### Urine Assay

In addition to standard laboratory tests, participants provide urine samples at their study visits (Table 1). These urine samples are analyzed using gas chromatography mass spectrometry to detect if HU is present. This method will be used to detect recent HU exposure and can detect HU in concentrations >1 µg/mL in the urine [32]. We will use this data to classify patients as adherent if they have levels >1 µg/mL and nonadherent if they have undetectable levels. Because HU adherence over multiple months is required to achieve a clinical benefit and this method can only determine if recent HU exposure has occurred, we will determine if intermittently detecting HU in the urine at study visits is correlated with video observed adherence.

#### Morisky Medication Adherence Scale, 4-Item

Participants complete the Morisky Medication Adherence Scale, 4-item (MMAS-4) at multiple time-points during the study (Table 1). The MMAS-4 is a validated, self-report adherence survey for adults that includes 4 yes/no questions [33-35], but it is unknown if this survey is valid in children with SCD.

#### Transition Readiness Assessment Questionnaire

Adolescents’ (16-19 years) self-management skills before and after receiving Mobile DOT are measured using the Transition Readiness Assessment Questionnaire (TRAQ) 5.0 (Table 1). The TRAQ 5.0 is a 20-item validated, patient-centered instrument that has 2 domains, self-management and self-advocacy [36].

#### Newest Vital Sign

Because health literacy has the potential to influence self-management [34], adolescent participants complete the newest vital sign (NVS) before and after receiving Mobile DOT (Table 1). The NVS is a validated survey that identifies patients at risk for low health literacy [37,38].

#### Satisfaction Survey

Participants complete the 5-point Likert scale Mobile DOT satisfaction survey that was created and modified during the pilot study to determine if participants found Mobile DOT intrusive, usable, and sustainable (Table 1). Participants are also asked to estimate the amount of time it takes to record the daily videos and to rate the importance of the incentives on their adherence behaviors.

#### Survey Completion

Participants complete all the surveys independently if they are ≥14-years old. Consenting caregivers of participants complete the surveys if the participant is <14. Surveys are completed electronically on an iPad and data is stored in a secure REDCap (REDCap Consortium) database.

### Clinical Outcomes

Participants’ electronic medical records are used to track the frequency of SCD-specific clinical outcomes (eg, vaso-occlusive pain episodes, acute chest syndrome episodes, or need for acute red cell transfusion) that were reduced with HU in the prior pediatric clinical trials [5-8].

### Study Withdrawal

Participants who do not send videos or respond to communications for more than 30 days during the Mobile DOT phase are withdrawn from the study because we are unable to confirm that they still have smartphone access and are receiving all of the Mobile DOT components. Participants are also able to voluntarily withdraw from the study at any time. Withdrawn participants complete a final study visit and we record the reason for withdraw (Table 1).

### Statistical Analysis

To determine if Mobile DOT improves HU adherence, we will compare the proportion of participants who achieve ≥80% HU adherence by MPR during the Mobile DOT phase with the proportion that achieved this level of adherence at baseline. This adherence level was chosen because improved clinical outcomes were seen when 90% of patients achieved this level of adherence during a large pediatric HU clinical trial [5,9]. We assume that we will have a 20% attrition rate and plan to enroll 72 participants to have 60 evaluable participants. This sample size will have 80% power, with an odds ratio of 6.869, using a one-sided McNemar test, and a significance level of .05.

We will use Spearman correlation coefficient to determine the correlation between video observed HU adherence and the other collected HU adherence measures (MMAS-4, MPR, MCV, HbF, and urine HU assay). Our sample size will have 80% power, at an alpha=.05, to detect a correlation coefficient ≥.35 between video adherence and the other adherence measures. To explore the impact of Mobile DOT on adolescents’ self-management skills, we will use McNemar test, and we will have 80% power to detect an effect size of 0.54, with a mean difference of 1 and standard deviation equal to 1.85, using a two-sided paired *t* test, with alpha=.05.

**Table 1:** Study design, visits, and data collection.

		Study phase							
		Baseline	Mobile DOT^a^			Observation		Off study
Study visit		No visit	1	2	3	4	5	Withdraw
Study day		−180 to 1	0	30-136	165-211	240- 316	345- 391	Anytime
**Data collection**								
	Demographic data		x					
	Hydroxyurea dose (mg/kg/d)	x	x	x	x	x	x	x
	Pharmacy list		x	x	x	x	x	x
	Medication possession ratio^b^	x			x		x	x
	Phone numbers		x	x	x	x	x	x
	Morisky Medication Adherence Scale, 4-item		x	x	x	x	x	x
	Satisfaction survey			x	x			x
	Newest vital sign^c^		x		x		x	x
	Transition Readiness Assessment Questionnaire 5.0^c^		x		x		x	x
	Laboratory data		x	x	x	x	x	x
	Urine assay		x	x	x	x	x	x
	Indication for withdraw							x

^a^Mobile DOT: Mobile Directly Observed Therapy.

^b^Medication possession ratio is calculated for each study phase and calculated after participants complete the study.

^c^Only for adolescent participants (those who are 16- to 19-years old) at enrollment.

## Results

**Figure 2:**
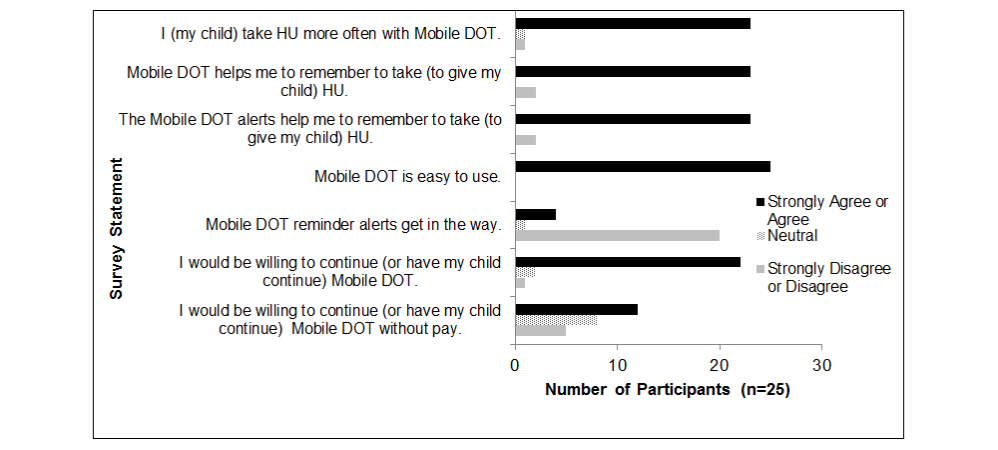
Preliminary satisfaction survey responses for participants that have completed the intervention phase. Mobile DOT: Mobile Directly Observed Therapy; HU: hydroxyurea.

This project was funded by the National Heart, Lung, and Blood Institute in September 2015. Participant enrollment is ongoing and we anticipate that all participants will have completed the study by early 2018. To date, we have approached 65 patients, enrolled 45, and 39 have either withdrawn (n=14) or completed their Visit 3 (n=25). Preliminary review of the satisfaction survey results show that most participants (n=38) complete Mobile DOT in less than 5 minutes each day. Most of the participants who completed the Mobile DOT phase of the study (n=25) also reported that they were satisfied with the intervention (Figure 2). Patients have withdrawn from the study because they either stopped HU or moved out of the country (n=2), stopped sending videos and responding to communications (n=6), had smartphone technical issues (n=1), lived in multiple homes that did not all have reliable smartphone access (n=3), or did not like receiving the text message alerts and did not have reliable smartphone access (n=2).

## Discussion

### Recruitment and Study Progress

We are successfully recruiting a large number of eligible patients. Similar to other feasibility studies of electronic interventions studies in minority populations [20], our preliminary satisfaction survey results suggest that participants who complete the intervention report that it improves their HU adherence. Our study attrition rate (31%) has been higher than anticipated. Because inconsistent access to a smartphone was a common reason for attrition in early participants, we have modified our eligibility criteria, which previously stated that participants had to “have access to a smartphone” to be more specific and specifically state that participants “have daily access to a smartphone” to limit future attrition.

### Future Implications

Despite demonstrated efficacy of HU, poor adherence is common among pediatric patients, and results in worse health outcomes [9-11]. Mobile DOT has the potential to fill an important gap in treating children with SCD by targeting specific adherence barriers for this patient population and multiple Health Behavior Model constructs. It leverages widely available smartphone technology to deliver a multifaceted adherence approach to a diverse patient population. If successful, it will increase HU adherence, which has the potential to improve patients’ clinical outcomes and reduce health care costs.

In addition, this study has the potential to inform other aspects of SCD-related patient care and the medication adherence field. First, determining the validity of other potential HU adherence measures will allow SCD providers and investigators to determine if routinely obtained (HbF and MCV), or easily obtained measures (MMAS-4 or a urine assay) can be used to identify patients with poor HU adherence and measure the effect of HU adherence interventions. Second, if Mobile DOT is successful in this complex patient population, this strategy could be tested in other patient populations where medication nonadherence is common and challenging. Lastly, exploring the effect that Mobile DOT has on adolescents’ self-management skills may inform future interventions to improve adolescent transition and result in improved health outcomes in these high-risk patients.

## Abbreviations

HbF: fetal hemoglobin
HU: hydroxyurea
MCV: mean corpuscular volume
MPR: medication possession ratio
Mobile DOT: Mobile Directly Observed Therapy
MMAS-4: Morisky Medication Adherence Scale
NCH: Nationwide Children's Hospital
NVS: newest vital sign
SCD: sickle cell disease
TRAQ: Transition Readiness Assessment Questionnaire
